# Two Novel Membranes Based on Collagen and Polyphenols for Enhanced Wound Healing

**DOI:** 10.3390/ijms252212353

**Published:** 2024-11-18

**Authors:** Victoria S. Shubina, Margarita I. Kobyakova, Nikita V. Penkov, Gennady V. Mitenko, Sergey N. Udaltsov, Yuri V. Shatalin

**Affiliations:** 1 Institute of Theoretical and Experimental Biophysics, Russian Academy of Sciences, Institutskaya 3, 142290 Pushchino, Russia; ritaaaaa49@gmail.com; 2 Institute of Cell Biophysics, Russian Academy of Sciences, Federal Research Center “Pushchino Scientific Center for Biological Research of the Russian Academy of Sciences”, Institutskaya 3, 142290 Pushchino, Russia; nvpenkov@rambler.ru; 3 Institute of Physicochemical and Biological Problems in Soil Science, Russian Academy of Sciences, Federal Research Center “Pushchino Scientific Center for Biological Research of the Russian Academy of Sciences”, Institutskaya 2, 142290 Pushchino, Russia; gen.mitenko@yandex.ru (G.V.M.); udaltsov@issp.serpukhov.su (S.N.U.)

**Keywords:** collagen-based materials, polyphenols, fibroblasts, wound, chemical burn

## Abstract

Two novel membranes based on collagen and two polyphenols, taxifolin pentaglutarate (TfG5) and a conjugate of taxifolin with glyoxylic acid (DfTf), were prepared. Fourier transform infrared spectroscopy examination confirmed the preservation of the triple helical structure of collagen. A scanning electron microscopy study showed that both materials had a porous structure. The incorporation of DfTf into the freeze-dried collagen matrix increased the aggregation of collagen fibers to a higher extent than the incorporation of TfG5, resulting in a more compact structure of the material containing DfTf. It was found that NIH/3T3 mouse fibroblasts were attached to, and relatively evenly spread out on, the surface of both newly obtained membranes. In addition, it was shown that the membranes enhanced skin wound healing in rats with a chemical burn induced by acetic acid. The treatment with the materials led to a faster reepithelization and granulation tissue formation compared with the use of other agents (collagen without polyphenols and buffer saline). It was also found that, in the wound tissue, the level of thiobarbituric acid reactive substances (TBARS) was significantly higher and the level of low-molecular-weight SH-containing compounds (RSH) was significantly lower than those in healthy skin, indicating a rise in oxidative stress at the site of injury. The treatment with collagen membranes containing polyphenols significantly decreased the TBARS level and increased the RSH level, suggesting the antioxidant/anti-inflammatory effect of the materials. The membrane containing TfG5 was more effective than other ones (the collagen membrane containing DfTf and collagen without polyphenols). On the whole, the data obtained indicate that collagen materials containing DfTf and TfG5 have potential as powerful therapeutic agents for the treatment of burn wounds.

## 1. Introduction

Burns remain one of the most serious health problems to date [1]. According to the World Health Organization, burns rank fourth among other traumas worldwide [1]. Patients with severe burns often need long-term hospitalization [2]. In some cases, the healing process may be incomplete, leading to changes in the structure of tissue and the loss of its function. The efficacy of healing depends on local factors such as the size and the depth of the wound, infection, etc. In addition, the healing process can be affected by other factors such as systematic immunological or nutritional deficiency, age, chronic comorbidities (diabetes, chronic venous insufficiency), etc. [3,4]. Thus, processes that occur during wound healing can differ substantially. For effective treatment of wounds, as complete an understanding as possible of the cellular and molecular mechanisms that drive tissue repair is required [3,5,6]. Refining the ideas about wound-healing mechanisms calls for changes in the requirements placed upon wound-healing materials. Currently, there are hundreds of wound-healing materials available on the market [7,8,9]. Nevertheless, in some cases, wound healing outcomes still remain insufficient [3] and must be improved. Therefore, the development of new wound-healing materials with desired properties, such as hemostatic, antibacterial, anti-inflammatory, and other properties, remains a research priority [3].

Materials based on collagen have received wide recognition in the treatment of wounds [10,11]. Collagen is the major structural protein of the connective tissue. It is produced by fibroblasts and plays an important role in the regulation of the wound healing process [12,13]. Collagen is a biocompatible and biodegradable biopolymer [11,13], which provides a biological scaffold for cell attachment, migration, and proliferation. Collagen dressings promote the deposition and organization of the newly formed collagen and support the formation of new tissues [14]. Nevertheless, the development of new collagen-based materials remains a focus of research [11,15]. Many investigations are aimed at improving the mechanical stability and reducing the biodegradation rate of collagen-based materials [11,15]. Other studies focus on the incorporation of biologically active compounds (antibiotics, anti-inflammatory compounds, regeneration stimulants, etc.) into the collagen matrices [10,16,17,18]. In this case, the collagen matrix was considered as a carrier for the targeted delivery of biologically active substances.

To improve the mechanical stability of the materials, the molecular structure of collagen is modified. Additional crosslinks are introduced using chemical, physical, and enzymatic crosslinking techniques. Among the crosslinking techniques, chemical crosslinking is the most effective [11]. It enables us to achieve a uniform and effective crosslinking [11]. In turn, the properties of crosslinked collagen materials are dependent on the chemical agent used [11]. In some cases, the crosslinker determines the negative effects of the materials. For example, crosslinking using glutaraldehyde, one of the oldest crosslinking agents whose molecule is retained in the crosslinked structure, can improve the mechanical properties of materials but results in the formation of cytotoxic compounds during degradation of the materials [19,20,21]. On the other hand, any changes in the material structure can influence the interaction of cells with materials. In particular, crosslinking using 1-ethyl-3-(3-dimethylaminopropyl)carbodiimide/N-hydroxysuccinimide (EDC/NHS), also known as a “zero-length” crosslinking agent, leads to the formation of crosslinks directly between the carboxyl groups of amino acid residues of glutamic/aspartic acids and the amino groups of amino acid residues of lysine of the polypeptide chains of the collagen molecules. Although materials obtained using this agent are more stable and biocompatible, the consumption of the carboxylate anion of glutamic acid, which is essential for cell attachment to collagen, causes a decrease in cellular spreading, survival, and growth [11,22]. To date, the search for new crosslinking agents has received much attention [11]. Of special interest are naturally occurring crosslinking agents due to their biocompatibility and low toxicity [11,23]. The available data in the literature demonstrate that polyphenols are capable of stabilizing the collagen structure and improving the mechanical properties of collagen-based materials through both noncovalent interactions (e.g., hydrogen bonding, electrostatic and hydrophobic interactions) and covalent polypeptide–polyphenol interactions [21,24,25,26,27,28,29]. Procyanidins [25,30], theaflavins [31], tannic acid [32,33,34], and other biologically active polyphenols [35] are considered as promising crosslinking agents.

It should be also noted that the materials containing biologically active compounds retain bioactivities, including antioxidant, antibacterial, and anti-inflammatory activities [15]. There is evidence indicating that polyphenols [36,37,38,39,40], their derivatives [41,42,43], and materials containing these compounds [44,45,46,47] improve wound healing. In particular, our previous results indicated that conjugates of taxifolin with carbonyl compounds and lipid peroxidation products such as acetaldehyde and malondialdehyde facilitated the regeneration of skin after chemical burn induced by acetic acid [43]. Taking the aforesaid into account, we propose that the incorporation into the collagen matrix of biologically active taxifolin derivatives containing in the structure one or more carboxyl groups would make it possible to stabilize collagen materials mainly through the formation of links between the carboxyl groups of polyphenols and the amino groups of lysine residues of collagen, without the involvement of the carboxylic groups of glutamic amino acid residues of the protein, which is critical to cell attachment to the material surface, and would promote wound healing.

**Figure 1:**
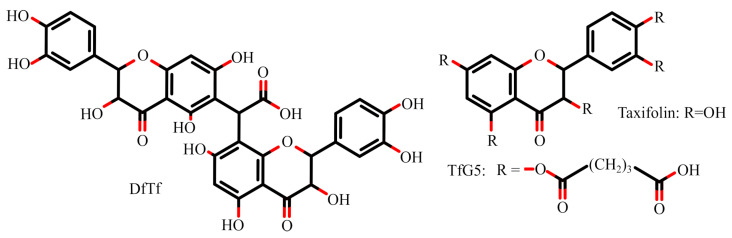
Structures of polyphenols used as collagen stabilizing agents. DfTf is a conjugate of taxifolin with glyoxylic acid, and TfG5 is taxifolin pentaglutarate.

The aim of this study was to prepare novel membranes based on collagen and polyphenols, a conjugate of taxifolin with glyoxylic acid (DfTf) and pentaglutarate of taxifolin (TfG5) (Figure 1), to characterize these materials using Fourier transform infrared spectroscopy (FTIR) and scanning electron microscopy (SEM), as well as to estimate their wound-healing properties.

## 2. Results

### 2.1. Characterization of the Membranes Based on Collagen and Polyphenols by FTIR

**Figure 2:**
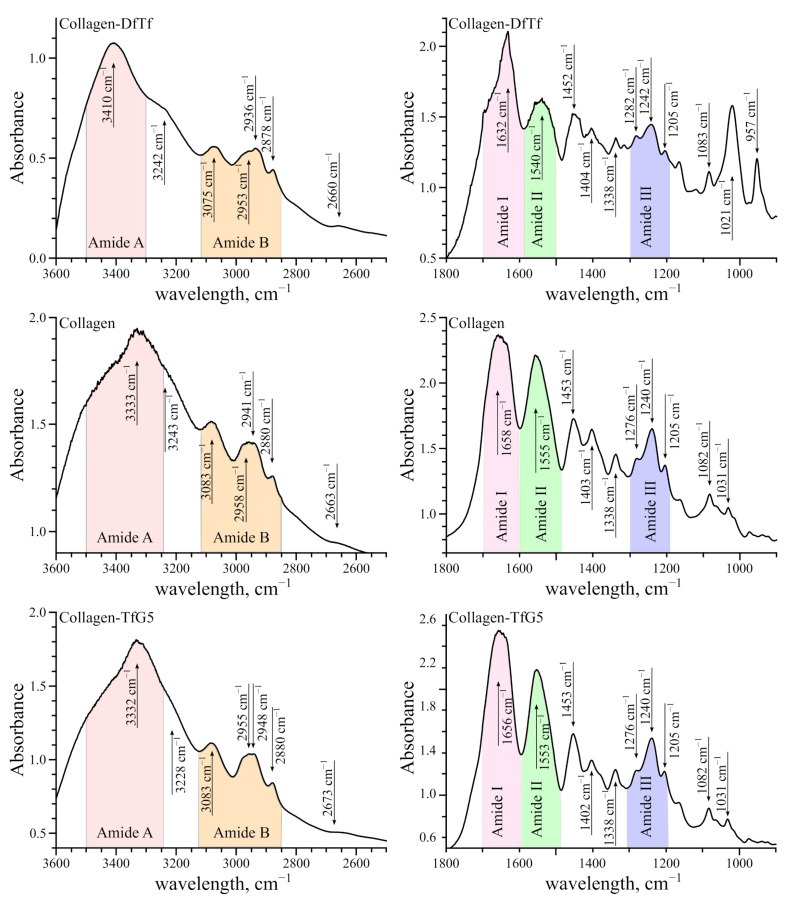
FTIR spectra of the materials based on collagen and taxifolin derivatives.

Figure 2 depicts the FTIR spectra of untreated collagen and collagen treated with polyphenols. All FTIR spectra of collagen showed bands characteristic for amide A (~3500–3300 cm^−1^), amide B (~3070 cm^−1^), amide I (~1700–1600 cm^−1^), amide II (~1600–1500 cm^−1^), and amide III (~1310–1175 cm^−1^) [48,49,50].

The amide A and B bands are generally assigned to the stretching vibrations of N–H groups (ν(N–H)) [49,51,52,53,54,55]. The amide I band is mainly associated with the stretching vibrations of C=O peptide groups (ν(C=O)) [48]. The amide II and amide III bands are generally attributed to the C-N stretching ν(C–N) and NH bending vibrations δ(N–H) [48,49]. In addition, all FTIR spectra exhibited absorption in the regions of 1350–1480 cm^−1^ (δ(CH_2_) and δ(CH_3_)) and 1005–1100 cm^−1^ (ν(C–O) and ν(C–O–C)).

The spectrum of untreated collagen showed the peak of amide A band at 3333 cm^−1^ and the peak of amide B band at 3083 cm^−1^. The peak of amide I band was observed at 1658 cm^−1^, the peak of amide II band at 1555 cm^−1^, and the peak of amide III band at 1240 cm^−1^; in addition, the peaks at 1453, 1403, 1338, 1276, 1205, 1082, and 1031 cm^−1^ were also recorded. The assignment of the selected bands can be found in SI Table S1 [48,49,53,54,55].

The spectrum of collagen treated with DfTf displayed the peak of amide A band at 3410 cm^−1^ and the peak of amide B band at 3075 cm^−1^. At the same time, the peaks corresponding to amide I, amide II and amide III were found at 1632, 1540 and 1242 cm^−1^, respectively. Compared to the spectrum of untreated collagen, the peaks of amide A and amide III bands shifted to higher wavenumbers (high frequencies), whereas the peaks of amide B, amide I, and amide II bands shifted to lower wavenumbers (low frequencies). The spectrum of DfTf-treated collagen also showed the peaks at 1452, 1404, 1338, 1282, 1205, and 1083 cm^−1^. New peaks appeared at 1702, 1120, 1021, 957, 714, 675, and 661 cm^−1^.

The FTIR spectrum of collagen treated with TfG5 had a number of peaks assigned to amide A, amide B, amide I, amide II, and amide III, which were located at 3332, 3083, 1656, 1553, and 1240 cm^−1^, respectively. Compared to the spectrum of untreated collagen, the peaks generally remained at the same positions. Nevertheless, some changes in the FTIR spectra were observed, e.g., a narrowing of the amide A band.

It should be noted that the ratio between the band of amide III (untreated collagen 1240 cm^−1^, DfTf 1242 cm^−1^, TfG5 1240 cm^−1^) and the bands at 1453 cm^−1^ (untreated collagen), 1452 cm^−1^ (DfTf), or 1453 cm^−1^ (TfG5) was approximately equal to 1.0, indicating that the triple helix structure of protein was preserved [50,56].

### 2.2. Characterization of the Membranes Based on Collagen and Polyphenols by SEM

**Figure 3:**
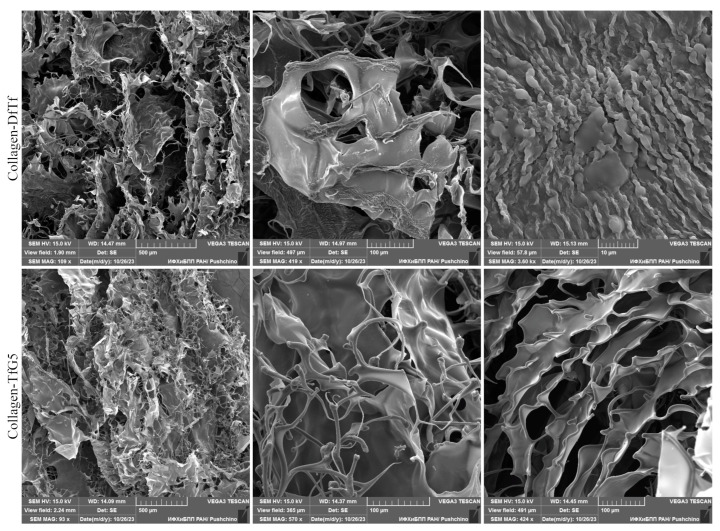
SEM images of the materials based on collagen and taxifolin derivatives.

Figure 3 shows the SEM images of the membranes based on collagen and the polyphenols (DfTf and TfG5). It can be seen that materials have a porous structure.

In certain areas of the materials, a network of thin cylindrical collagen fibers was found. The network of such fibers in the material containing TfG5 was more extensive than in the material containing DfTf, indicating that the incorporation of DfTf into the material increases the aggregation of collagen fibers to a higher extent than the incorporation of TfG5, resulting in a more compact structure of the material containing DfTf.

### 2.3. Attachment and Spreading of NIH/3T3 Fibroblasts on the Surface of the Collagen-Based Membranes Containing Polyphenols

**Figure 4:**
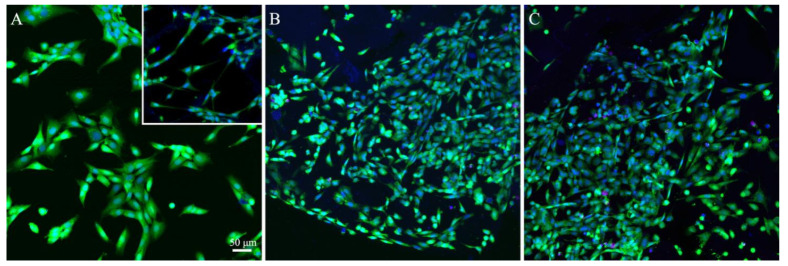
Confocal laser scanning microscopy images of NIH/3T3 fibroblasts cultured for 24 h on the surface of the collagen materials containing taxifolin derivatives. (**A**) The control. The cells were seeded on the surface of a collagen matrix without polyphenol. (**B**) The collagen materials containing DfTf. The cells were seeded on the surface of the collagen material containing 2.5% DfTf. (**C**) The collagen materials containing TfG5. The cells were seeded on the surface of the collagen material containing 2.5% TfG5. Cell nuclei were stained with Hoechst 33,342 (live and dead cells; seen in blue) and propidium iodide (dead cells; seen in red). The cytoplasm of live cells was stained with calcein-AM (seen in green). Scale bar: 50 μm.

It was found that collagen that coated culture dishes almost completely degraded during cultivation for 24 h. Fibroblasts attached to the dish surface and spread on it (Figure 4A). In some areas, small fragments of collagen gel were observed, and fibroblasts also spread on the surface of these fragments (Figure 4A, insert). The materials containing polyphenols were significantly more stable than the matrices made of collagen solely. The specimens were transferred to a glass slide and examined under a confocal microscope. It was found that fibroblasts attached to the surface of the membranes and spread on it (Figure 4B,C). No toxic effect of the membranes on the cells was revealed.

### 2.4. Wound Closure

**Figure 5:**
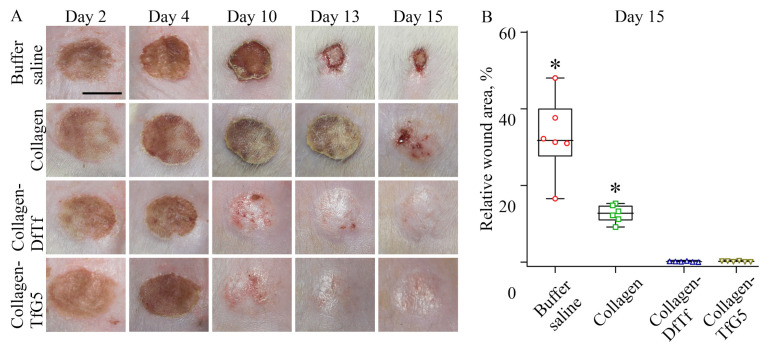
The effects of the collagen materials containing polyphenols on wound healing. (**A**) Representative photographs of wounds treated with the materials at different time points after wounding. (**B**) The relative wound area treated with the materials on day 15 after injury. The data were analyzed using Mann–Whitney U-test. * *p* < 0.05 compared to other groups.

To evaluate the effect of the membranes on tissue regeneration, a model of chemical burn induced by acetic acid was used. To assess wound healing progress, the photographs of wounds were taken every day until one group was completely healed. Representative photographs of the wounds at different time points are presented in Figure 5.

It can be seen that a scab formed over the wound. In control groups, the scab covered the wound up to the 15th day post-wounding. In the experimental groups, the scab fell off within 10 to 13 days post-wounding. Under the scab, reepithelization was observed. Planimetric measurements showed that the wound areas in the experimental groups (collagen-TfG5 and collagen-DfTf) were smaller than those of the control groups (buffer saline and collagen) from the 10th–13th day after burn injury.

Figure 5 shows wound areas in the groups on the 15th day. Thus, the data indicate that the wound closure was more intensive in the experimental groups than that in the control groups.

### 2.5. Histological Examination

**Figure 6:**
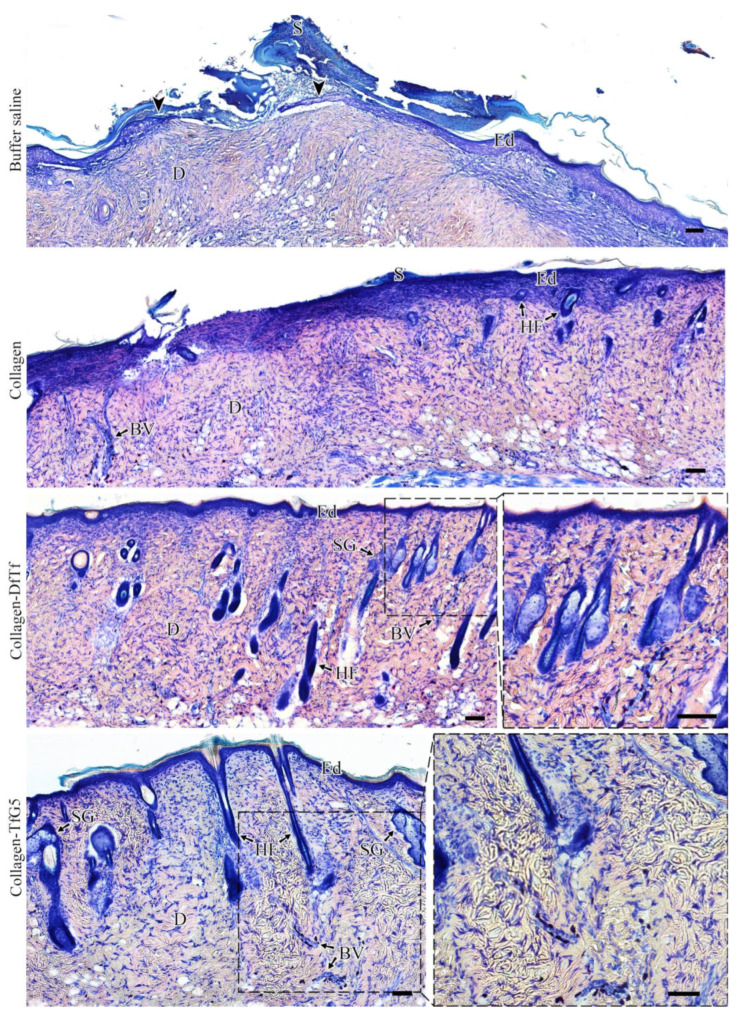
Histological evaluation of the effect of the collagen materials containing taxifolin derivatives on wound healing on day 15 after injury. Histological sections were stained with azure and eosin (abbreviations used: S, scab; Ed, epidermis; D, dermis; BV, blood vessel; HF, hair follicle; SG, sebaceous gland). Arrowheads indicate epithelization edges. Scale bar: 100 μm.

Figure 6 shows representative tissue sections from the wound sites collected on day 15. In the saline-treated group, the wound was covered with a scab. Incomplete reepithelization and granulation tissue formation were observed. In the group treated with collagen, the reepithelization of the wounds was faster than that in the saline-treated group. Nevertheless, the reepithelization was incomplete, and scab fragments were still found (Figure 6). At the same time, new blood vessels and hair follicles were observed. In the experimental groups (collagen-TfG5 and collagen-DfTf), the scab was absent, and reepithelization was complete. New blood vessel, hair follicles, and sebaceous glands were observed. Thus, the treatment of wounds with the collagen-based membranes containing polyphenols promotes more intensive wound healing compared to the control (the treatment with collagen and buffer saline).

### 2.6. Levels of Thiobarbituric Acid Reactive Substances and Low-Molecular-Weight SH-Containing Compounds in the Tissue

The first stage of wound repair is inflammation [57]. During this stage, neutrophils produce reactive oxygen species (ROS), which contribute to lipid peroxidation. Here, the level of thiobarbituric acid reactive substances (TBARS), a marker of lipid peroxidation [58], was estimated at the site of injury. The level of low-molecular-weight SH-containing compounds (RSH), which reflects the antioxidant capacity of the wound tissue [59], was also assessed.

TBARS and RSH levels were estimated on the fourth and eighth days post-wounding. It was found that, in a wound tissue, the level of TBARS was significantly higher and the level of RSH was significantly lower than those in the healthy skin, indicating a rise in oxidative stress in the injured tissue (Table 1). The treatment of wounds with collagen membranes (with and without polyphenols) resulted in a significant decrease in the tissue level of TBARS compared to the treatment of wounds with buffer saline (the negative control group). On the fourth day, the level of TBARS in the groups treated with the collagen-based membranes containing polyphenols was lower than that in the group treated with collagen only. The collagen-based membrane containing TfG5 was more effective than the other membranes. On the eighth day, the level of TBARS was significantly lower in the groups treated with collagen membranes (with and without polyphenol) than that in the negative control group treated with buffer saline and was approaching the level of TBARS in the normal control group (healthy skin). The level of RSH was significantly higher in the groups treated with collagen membranes (with and without polyphenol) than that in the negative control group treated with buffer saline. In the groups treated with the membranes containing polyphenols, the level of RSH was higher than that in the group treated with collagen. Thus, the data obtained suggest antioxidant/anti-inflammatory properties of the polyphenols.

## 3. Discussion

Our previous results indicate that, on the basis of collagen and the taxifolin derivatives (DfTf and TfG5), nontoxic and stable to degradation materials can be prepared [60,61]. Thus, we earlier showed that NIH/3T3 mouse fibroblasts migrate through these materials. Noteworthily, an increase in the proportion of DfTf in the material inhibited the migration through it, whereas an increase in the proportion of TfG5, on the contrary, significantly increased cell migration through the material. Further confocal microscopy studies of the materials showed that, despite the complete absence of cell migration through the gel containing 2.5% DfTf, fibroblasts attached to the surface of the material but did not spread out on it. At the same time, fibroblasts attached to, and spread out on, the surface of the material containing 2.5% TfG5, the migration through which exceeded the migration through native collagen by twofold [60]. The cells penetrated deep into the gel material and spread out within the three-dimensional matrix (Supplementary Video S1). It was proposed that a decrease in cell spreading on the surface of the material containing DfTf, as well as a decrease in the migration of the cells through the gel with increasing proportion of DfTf in the material, are related, at least partially, to a higher involvement of the functional groups of amino acid residues of collagen in the interactions with DfTf. Taking the aforesaid into account, we modified the technique of crosslinking of collagen by polyphenols. Previously, a solution of a polyphenol was directly added to a solution of collagen (4%), after which the mixture was stirred and poured into the prepared molds. In the frame of the present work, a solution of a polyphenol was added to a collagen sponge, which was preliminary prepared by the lyophilization of a collagen solution. We suggested that the spatial arrangement of collagen molecules relative to each other in a solution differ from that in the sponge; therefore, the set of functional groups available for interactions with a polyphenol molecule in solution differ from that in the sponge. Accordingly, the sets of functional groups available for the interactions with cells within the materials prepared using different procedures also differ.

FTIR spectra of untreated collagen and collagen treated with polyphenols showed characteristic bands for amide A, amide B, amide I, amide II, and amide III. The spectrum of collagen treated with DfTf demonstrated that the amide A band is shifted to higher frequencies (compared to untreated collagen), suggesting that the NH groups were involved in a new set of hydrogen bonds, probably, with polyphenolic compounds [62]. The shift of amide B and amide II bands to low frequencies also counts in favor of this suggestion [62]. The FTIR spectrum of collagen treated with TfG5 showed that the peaks remained predominantly at the same positions. However, some changes in the FTIR spectra were still observed. For instance, a narrowing of the amide A band was found. In order to estimate the preservation of the triple helix, the FTIR absorption ratio of amide III to 1452 cm^−1^ band (AIII/A1452) was determined. This ratio was about 1.0 for all samples being tested (untreated collagen, DfTf-treated collagen, and TfG5-treated collagen), indicating that the triple helix structure was preserved [50,56].

SEM data indicated that both materials had a porous structure. In some areas of the membranes, a network of thin cylindrical collagen fibers was observed. The network of these fibers was more extensive in the membrane containing TfG5 than in the DfTf-treated collagen, indicating that the incorporation of DfTf into the material increases the aggregation of collagen fibers to a higher extent than the incorporation of TfG5, which results in a more compact structure of the material containing DfTf. Similar morphological changes of collagen materials were observed after the treatment of the materials with crosslinking agents. In particular, glutaraldehyde crosslinking increased the aggregation of collagen fibers and resulted in a more compact structure of the materials [63]. The increase in the glutaraldehyde concentration and/or the reaction time led to a higher aggregation of collagen fibers and a more compact structure [63]. It is remarkable that the data on the stability of gel materials obtained by mixing a collagen solution with solutions of the polyphenols showed that the materials containing DfTf were degraded more slowly than those containing TfG5 at the same concentration [60]. On the whole, these data suggest that the functional groups of collagen are involved in the interactions with DfTf to a higher extent than in the interactions with TfG5.

Confocal microscopy studies showed that fibroblasts were attached to the surface of both membranes and spread on it. As in previous studies [60], no toxic effect of the materials on cells was found.

In line with confocal microscopy observations (good fibroblasts attachment and spreading on the surface of the two newly obtained collagen–polyphenols systems showing no cytotoxicity), the experimental findings suggest a useful practical approach to obtaining stable and noncytotoxic collagen-DfTf/TfG5 products with different properties largely influenced by changing the initial preparation conditions.

Generally, numerous studies regarding the importance of biologically active polyphenols as molecular compounds in stabilizing the native structure of collagen have been reported [54,64,65,66,67,68]. In particular, it was found that DfTf and TfG5 exhibited high antioxidant activity in cell-free systems [64,65,66]. It was also shown that these polyphenols can modulate the functions of neutrophils [64,67,68]. Neutrophils, as known, are essential modulators of inflammatory and immune responses and play an important role in wound repair [69,70,71]. It was found that DfTf and TfG5 inhibited the production of ROS by neutrophils stimulated with phorbol 12-myristate 13-acetate (PMA) [64,68]. DfTf inhibited the phagocytosis of latex beads opsonized with IgG by neutrophils but significantly enhanced the adhesion of these cells to the plastic surface during phagocytosis [68]. Thus, on the one hand, the inhibitory effects indicate the anti-inflammatory potential of both compounds. On the other hand, enhanced adhesion of neutrophils to the plastic surface in the presence of DfTf counts in favor of the possible proinflammatory action of this polyphenol. For instance, the adhesion of neutrophils is associated with the secretion of various cytokines that promote tissue infiltration by macrophages and the maintenance of the inflammatory process [69,72,73,74]. At the same time, some of these cytokines, in particular interleukin-1β, activate fibroblast-like cells [70], which are known to be involved in the process of tissue regeneration [75].

The evaluation of the effect of the membranes prepared on the regeneration of skin tissue after chemical burn showed that they enhance wound healing. The collagen–polyphenol-containing materials accelerate reepithelization and granulation tissue formation. These results are in good agreement with the data in the literature, according to which flavonoids [37,38,39], their derivatives [41,42,43], and materials containing these compounds [44,45,47] improve wound healing. In particular, a faster wound contraction was shown in the groups treated with quercetin-incorporated collagen films [44] and curcumin-incorporated collagen matrix [45] compared to the control (the treatment with collagen alone). Enhanced wound healing was observed in diabetic mice treated with an epigallocatechin gallate-incorporated collagen sponge [76]. Collagen films treated with procyanidin B from a *Cassia auriculata* leaf extract also showed promising results in wound healing [47]. The plant extract also improved the physical properties of collagen films [47].

However, it should be noted that ambiguous results exist regarding the action of some polyphenols on wound healing [77,78,79]. For example, Park and coauthors found that quercetin can have a detrimental effect on burn wound healing [78], whereas Gouma and coauthors showed a beneficial effect of this flavonoid [79]. Probably, this may be due to different experimental conditions and assessment methods used by the authors. Zhao and coauthors showed that chronic topical administration of resveratrol accelerated wound healing in young rats, while its intermittent administration did not [77]. These data suggested that the effects of this compound on wound healing were dependent on persistent usage [77]. Nevertheless, no obvious effect on wound healing in aged rats was observed after chronic applications of resveratrol. At the same time, it was shown that resveratrol stimulates AMP-activated protein kinase (AMPK), a key mediator of wound healing, and improves the vascularization of wound beds, indicating that this polyphenol has remarkable potential in improving the regeneration of aged skin. The authors proposed that varying the experimental conditions, e.g., the concentration of resveratrol or the period of observation, may prove the therapeutic effect of this compound on aged wounds [77].

It should be noted that an increase in the concentration of polyphenols does not always lead to a beneficial effect on wound healing [76,80]. In particular, Kim and coauthors showed that the treatment of wounds with a collagen sponge incorporating epigallocatechin-3-O-gallate at a low concentration (10 ppm) enhanced wound healing in diabetic mice [76]. In contrast, a delayed wound closure was observed in mice treated with a sponge incorporating polyphenol at a concentration of 1000 ppm. The wound closure in mice treated with a sponge containing polyphenol at a concentration of 100 ppm was comparable to the control (collagen sponge) [76]. Kant and coauthors showed that a topical application of 0.1% quercetin caused the fastest wound closure, as compared to other treatments (1.0 and 10.0%) [80].

Interesting data were reported by Yeh and coauthors [42]. It was shown that artocarpin (ARTO), a prenylated flavonoid purified from the plant *Artocarpus communis*, enhanced skin wound healing. It is remarkable that, on day 1 after injury, the numbers of neutrophils and macrophages in the wound area were higher in the ARTO-treated group than in the control group. However, on day 3, there were fewer neutrophils and macrophages in the ARTO-treated group than in the control group. Thus, the data obtained by the authors suggest that APTO accelerates the inflammatory phase progression by causing an early peak of inflammation with accelerated infiltration and elimination of inflammatory cells, followed by a decrease in persistent inflammation [42].

Here, we showed that, in the wound tissue, the level of TBARS was significantly higher and the level of RSH was significantly lower than those in the healthy skin, indicating a rise in oxidative stress in the injured tissue. The treatment with collagen membranes (with and without polyphenols) led to a significant decrease in the TBARS level and an increase in the RSH level. Polyphenol-containing collagen membranes were more effective than membranes made of collagen alone, suggesting an antioxidant/anti-inflammatory effect of polyphenols. These results are consistent with the data in the literature, according to which the treatment of burns with a cream containing isoquercetin led to a significant decrease in the TBARS level and an increase in the GSH level in the burn wound tissue, as well as a significant increase in the percentage of wound contraction in comparison with the control (the burn injury) [81].

In general, the results of this study indicate that collagen materials containing polyphenols promote wound closure and accelerate reepithelization and granulation tissue formation. Probably, polyphenols can influence the regeneration process during three sequential and overlapping phases of regeneration (the inflammatory, proliferative, and remodeling phases [57]). This suggestion is supported by the data in the literature, according to which polyphenols can modulate the functions of various cells types, including immune cells, fibroblasts, and endothelial cells [38,82]. Some of these compounds can influence the activation of intracellular signaling pathways, such as NF-kB, MAPK, and JAK-STAT [38,82,83]. The influence of polyphenols on Ras/Raf/MEK/ERK, PI3K/Akt, and NO pathways has also been reported [38,82,83]. Thus, the data suggest that collagen materials containing polyphenols enhance wound healing, possibly, through various mechanisms of action. Collagen materials containing DfTf and TfG5 may be considered as materials that have a dual purpose: they enhance wound healing and deliver drugs to the site of injury. On the whole, the data obtained indicate that collagen materials containing DfTf and TfG5 has potential as potent therapeutic agents for the treatment of burn wounds.

**Figure 7:**
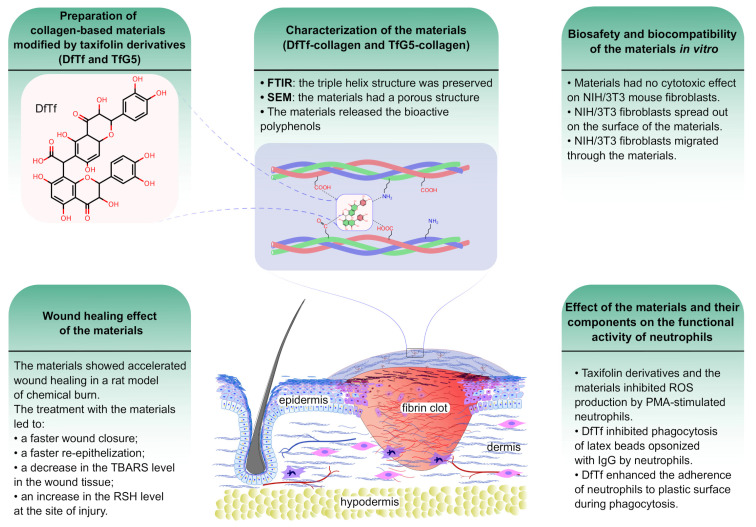
A schematic diagram of the properties of the collagen materials containing taxifolin derivatives. The release of the polyphenols from the materials [60], the migration of fibroblasts through the materials [60], and the effect of the materials and their components on the functional activity of neutrophils [60,67] have been previously studied.

The summarized data on the properties of the collagen materials containing DfTf and TfG5 are given in Figure 7.

## 4. Materials and Methods

### 4.1. Materials

Collagen (from bovine skin) was purchased from CJSC “Zelenaya Dubrava” (Dmitrov, Russia). Taxifolin was kindly provided by Flavit (Pushchino, Russia). All reagents were of analytical grade. Water used for the preparation of solutions was purified using a Milli-Q system (Millipore, Burlington, MA, USA).

### 4.2. Preparation of Collagen Membranes

Collagen was dissolved in 0.2 M acetic acid at a concentration of 4.0%. Dry collagen (400 mg) was added to 10 mL of acetic acid (0.2 M), and the mixture was placed in an incubator at 25 °C with orbital shaking at 150 rpm until the collagen was completely dissolved (~12 h). Then, collagen solution was poured into cylindrical molds with a diameter of 1.5 cm and a volume of 1 mL. After that, the collagen samples were freeze-dried. In order to remove residual acetic acid, collagen scaffolds were washed three times with ice-cold water (20 mL). Then, the samples were freeze-dried again. After that, the samples were treated with polyphenols (DfTf and TfG5) or remained untreated (collagen without polyphenols).

### 4.3. Preparation of Polyphenol-Incorporated Collagen Membranes

To activate the carboxylic groups of polyphenols, 4-(4,6-Dimethoxy-1,3,5-triazin-2-yl)-4-methylmorpholin-4-ium chloride (DMTMM) was used. Reaction mixtures (20 mL) contained NaH_2_PO_4_/Na_2_HPO_4_ (20 mM, pH 7.4), polyphenol (40 mg), and DMTMM (100 mg). The mixtures were stirred for one hour; after that, solutions containing polyphenols at different concentrations (2.5%, 1.0%, and 0.5%) were prepared. Then, 1 mL of the polyphenol solution was added to the collagen scaffold. The samples were then placed in a thermal shaker and incubated at 37 °C for 24 h. After that, the samples were washed three times with PBS and freeze-dried.

### 4.4. Characterization of the Samples

The samples for the FTIR study were prepared in the form of KBr-based pellets. To achieve this, 700 mg of KBr FT-IR grade powder (Sigma-Aldrich, St. Louis, MO, USA) was mixed with 15 mg of the analyzed substance, ground in an agate mortar, and pressed into pellets with a diameter of 13 mm at a pressure of 5 kbar for 1 min. A 700 mg pellet of pure KBr was also prepared to record the background spectrum. The absorption spectra of the pellets were measured using a Nicolet 6700 spectrometer (Thermo, Waltham, MA, USA) with a resolution of 2 cm^−1^ in the wavenumber range of 400–4000 cm^−1^. The DTGS detector, IR radiation source, and KBr beam splitter were used. Interferograms were processed by a standard software of the spectrometer using Happ–Genzel apodization function and ×4 zero filling. The thickness of the pellets was about 2 mm, which for KBr with a refractive index of 1.54 corresponds to the etalon effect with a band spacing of about 1.7 cm^−1^. Since the selected spectral resolution was higher (2 cm^−1^) and apodization was applied, the etalon effect did not appear in the spectra. After placing the samples in the sample compartment, a pause of 1 min was maintained before measurement to purge the cuvette compartment with air with a reduced content of water vapor and CO_2_ using an FT-IR Purge Gas Generator 74-5041 (Parker Hannifin Corporation, Haverhill, MA, USA).

The morphology of the samples was studied using a scanning electron microscope (SEM; TESCAN Vega 3 LSU, Brno, Czech Republic). The samples were covered with gold before the analysis.

### 4.5. Cell Culture

Mouse embryonic fibroblast NIH/3T3 cells were obtained from the Russian Cell Culture Collection (Institute of Cytology of the Russian Academy of Sciences, St. Petersburg, Russia). The cells were grown in DMEM/F-12 medium (Sigma-Aldrich, USA) supplemented with 10% fetal bovine serum (FBS) (Himedia, Maharashtra, India), 80 μg/mL gentamicin sulfate (Sigma-Aldrich), and 24 μg/mL fluconazole (Belmedpreparations, Minsk, Belarus) at 37 °C and 5% CO_2_. A solution of trypsin–EDTA 0.05% (PanEco, Moscow, Russia) was used to detach cells from the surface of the culture plastic.

### 4.6. Attachment and Spreading of NIH/3T3 Fibroblasts on the Surface of the Membranes Obtained

Membranes containing 2.5% polyphenol were prepared in 35 mm Petri dishes. The cells (1.5 × 10^4^ cells/cm^2^) were seeded on their surface and incubated for 24 h. The samples were stained for 30 min with Hoechst 33,342 (1 μg/mL; Sigma-Aldrich), propidium iodide (1 μg/mL; Sigma-Aldrich), and calcein acetoxymethyl ester (calcein-AM, 100 nM; Sigma-Aldrich) and were studied using a TCS SP5 confocal microscope (Leica Microsystems, Wetzlar, Germany). Collagen that coated the surface of Petri dishes was used as a control.

### 4.7. Wound Healing

The wound-healing effect was estimated on a rat model of chemical burn. All animal experiments were carried out in compliance with the Directions of the European Parliament 2010/63/EC and were approved by the Ethics Committee (Institute of Theoretical and Experimental Biophysics, Russian Academy of Sciences; Permission № 23 of 18 March 2024). A total of 44 adult male Wistar rats weighing between 230 and 260 g were used. The animals were anesthetized with Zoletil^®^ 100 (Virbac, Carros, France) in combination with Rometar^®^ (2% xylazine, Bioveta, a.s., Ivanovicena Hané, Czeck Republic). The dose of the components was 12.5 mg/kg (for zolazepam) and 7.5 mg/kg (for xylazine).

One day before the experiment, the dorsal areas of the rats were depilated. Then, a round skin wound with a diameter of 7.5 mm was created on the back of each rat. For that, gauze soaked with glacial acetic acid was placed on depilated skin for 60 s. After that, the rats were randomized into four groups (eleven rats per group): (1) the negative control group treated with buffer saline; (2) the control group, which received collagen without polyphenols; (3) the group treated with collagen material containing DfTf (collagen-DfTf); (4) the group treated with collagen material containing TfG5 (collagen-TfG5).

The collagen materials or buffer saline were administered daily to each rat by topical application until one group was completely healed. To evaluate wound healing progress, the photographs of wounds were taken every day. Wound areas were measured with ImageJ software (Version 1.54f), and the values were calculated as follows: wound area (%) = (wound area on a particular day/wound area on day 0) × 100%.

### 4.8. Wound Histology

The rats were euthanized on day 15 and the wound skin samples were collected and fixed in 4% paraformaldehyde, embedded, and sectioned for histological analysis. The wound skin samples were cut into 10 μm thick sections, which were stained with azure and eosin mixture (Minimed, Bryansk, Russia).

### 4.9. Levels of TBARS and RSH in the Tissue

Skin tissue samples (~100 mg) were homogenized (*w*/*v* 10%) in 10% trichloroacetic acid and centrifuged at 10,000 rpm at 4 °C for 5 min. The supernatants obtained were used for determining TBARS and RSH levels. The level of TBARS was examined by monitoring the reaction between lipid peroxidation products and the thiobarbituric acid [84]. The supernatant (0.1 mL) was treated with 2 mL of the mixture containing thiobarbituric acid (0.67%), HCl (0.25 N), and trichloroacetic acid (10%). Then, the mixture was kept in a water bath for 15 min and further cooled and centrifuged for 10 min at room temperature. Absorbance was measured at 532 nm, and the results were expressed as nmol TBARS/g-tissue. The level of RSH was evaluated using Ellman’s reagent [85]. The supernatant (1.0 mL) was mixed with 0.5 mL of Ellman’s reagent (0.5 mM) and 3.0 mL of phosphate buffer (20 mM, pH 7.4). Absorbance was measured at 412 nm, and the results were expressed as μmol RSH/g-tissue. The amount of sulfhydryls in the sample was determined using the molar extinction coefficient of 2-nitro-5-thiobenzoic acid (14,150 M^−1^ cm^−1^) [85].

### 4.10. Statistical Analysis

Wound healing study: Group comparisons were performed using the Mann–Whitney U-test. *p* < 0.05 was considered statistically significant. The estimation of TBARS and RSH levels: The data are given as the mean ± standard deviation. Differences between groups of data were analyzed by one-way analysis of variance (ANOVA) followed by Tukey’s test (GraphPad Prism 9.0.0, GraphPad Software Inc., San Diego, CA, USA). Differences with a *p* value < 0.05 were considered statistically significant.

## Figures and Tables

**Table 1:** Levels of TBARS and RSH in the tissue.

Group	TBARS, nmol/g-tissue		RSH, µmol/g-tissue	
	4th Day	8th Day	4th Day	8th Day
Control (negative control group)	13.91 ± 0.09 *	10.85 ± 0.05 *	1.41 ± 0.06 *	2.18 ± 0.07 *
Collagen	10.84 ± 0.11 *	8.40 ± 0.22	1.95 ± 0.10 *	2.84 ± 0.09 *
Collagen-TfG5	8.86 ± 0.09 *	7.87 ± 0.23 ^#^	2.17 ± 0.11 *	3.31 ± 0.10
Collagen-Df-Tf	9.51 ± 0.16 *	8.11 ± 0.10	2.01 ± 0.10 *	3.17 ± 0.10
Healthy skin (normal control group)	7.33 ± 0.02		3.28 ± 0.09	

The values are the means ± standard deviation. * Differences are significant compared to other groups *p* < 0.05. ^#^ Differences are significant compared to groups treated with the collagen material without polyphenols and the material containing DfTf, *p* < 0.05.

## Data Availability

The data presented in this study are available upon request from the corresponding author.

## Supplementary Materials

The following supporting information can be downloaded at: https://www.mdpi.com/article/10.3390/ijms252212353/s1.
