# Supplementary material for: Two Novel Membranes Based on Collagen and Polyphenols for Enhanced Wound Healing
Source: Int J Mol Sci. 2024 Nov 18;25(22):12353. doi: 10.3390/ijms252212353 (PMC11594507; doi:10.3390/ijms252212353)
Supplement: Supplementary file 1 [file ijms-25-12353-s001.zip › SI_Table 1.pdf]

International Journal of *Molecular Sciences*

MDPI

Supplementary materials

Two Novel Membranes Based on Collagen and Polyphenols for Enhanced Wound Healing

Victoria S. Shubina [1,*,†], Margarita I. Kobyakova [1], Nikita V. Penkov [2], Gennady V. Mitenko [3], Sergey N. Udaltsov [3] and Yuri V. Shatalin [1,*,†]

1 Institute of Theoretical and Experimental Biophysics, Russian Academy of Sciences, Institutskaya 3, 142290 Pushchino, Russia; ritaaaaa49@gmail.com
2 Institute of Cell Biophysics, Russian Academy of Sciences, Federal Research Center "Pushchino Scientific Center for Biological Research of the Russian Academy of Sciences", Institutskaya 3, 142290 Pushchino, Russia; nvpenkov@rambler.ru
3 Institute of Physicochemical and Biological Problems in Soil Science, Russian Academy of Sciences, Federal Research Center "Pushchino Scientific Center for Biological Research of the Russian Academy of Sciences", Institutskaya 2, 142290 Pushchino, Russia; gen.mitenko@yandex.ru (G.V.M.); udaltsov@issp.serpukhov.su (S.N.U.)
* Correspondence: shubinavs@iteb.pushchino.ru (V.S.S.); shatalinyuv@iteb.pushchino.ru (Y.V.S.)
† These authors contributed equally to this work.

Table S1. The assignment data for the collagen materials containing polyphenols.*

Region	Wavenumber (cm−1)			Characteristic
	Collagen	Collagen-DfTf	Collagen-TfG5	
Amide A	3333	3410	3332	ν(N-H) [1-3]
Amide B	3083	3075	3083	ν(N-H) [1,2]
	2958	2953	2955	ν(C-H) [1]
	2941	2936	2948	ν(C-H) [1], ν(CH_2) [1,2]
	2880	2878	2880	ν(C-H)[1], ν(CH_3) [1,2], ν(CH_2) [3]
Amide I	1658	1632	1656	ν(C=O) [1-3]
Amide II	1555	1540	1553	ν(C−N) [1-3], δ(N−H) [1-3]
Amide II [3]	1453	1452	1453	δ(CH2) [1,2,3], δ(CH3) [1]
Amide II [3]	1403	1404	1402	$ν_s$(COO-)
	1338	1338	1338	δ(CH2) δ(N-H) ν(C-N) [1],
Amide III	1240	1242	1240	ν(C−N) [1-3], δ(N−H) [1,2], $γ_w$(CH_2) [2,3]
	1205	1205	1205	δ(N-H) [1], ν(C-N) [1]
Cbh [1,2]	1082	1083	1082	ν(C-O) [1,2,3], ν(C-O-C) [2], ν(C-N-C) [3]
Cbh [1,2]	1031		1082	ν(C-O) [1,3], ν(C-O-C) [2]

* Some vibrational bands are strongly affected by the overlapping with some other bands [4]. In addition, some bands have several main peaks [4]. Abbreviations used: Cbh - carbohydrates.

References

1. Martínez Cortizas, A.; López-Costas, O. Linking Structural and Compositional Changes in Archaeological Human Bone Collagen: An FTIR-ATR Approach. Scientific Reports 2020, 10, doi:10.1038/s41598-020-74993-y.

2. L. Brăzdaruet et al. 3D Porous Collagen Matrices-A Reservoir for In Vitro Simultaneous Release of Tannic Acid and Chlorhexidine. 2022. Pharmaceutics. 15(1):76. doi: 10.3390/pharmaceutics15010076.

3. H. Ju, et al. Comparison of the Structural Characteristics of Native Collagen Fibrils Derived from Bovine Tendons using Two Different Methods: Modified Acid-Solubilized and Pepsin-Aided Extraction. 2020. Materials (Basel), 13(2):358. doi: 10.3390/ma13020358.

4. Stani, C.; Vaccari, L.; Mitri, E.; Birarda, G. FTIR Investigation of the Secondary Structure of Type I Collagen: New Insight into the Amide III Band. Spectrochimica Acta Part A: Molecular and Biomolecular Spectroscopy 2020, 229, 118006, doi:10.1016/j.saa.2019.118006.
