# Supplementary figures and images for: Two Novel Membranes Based on Collagen and Polyphenols for Enhanced Wound Healing
Source: Int J Mol Sci. 2024 Nov 18;25(22):12353. doi: 10.3390/ijms252212353 (PMC11594507; doi:10.3390/ijms252212353)

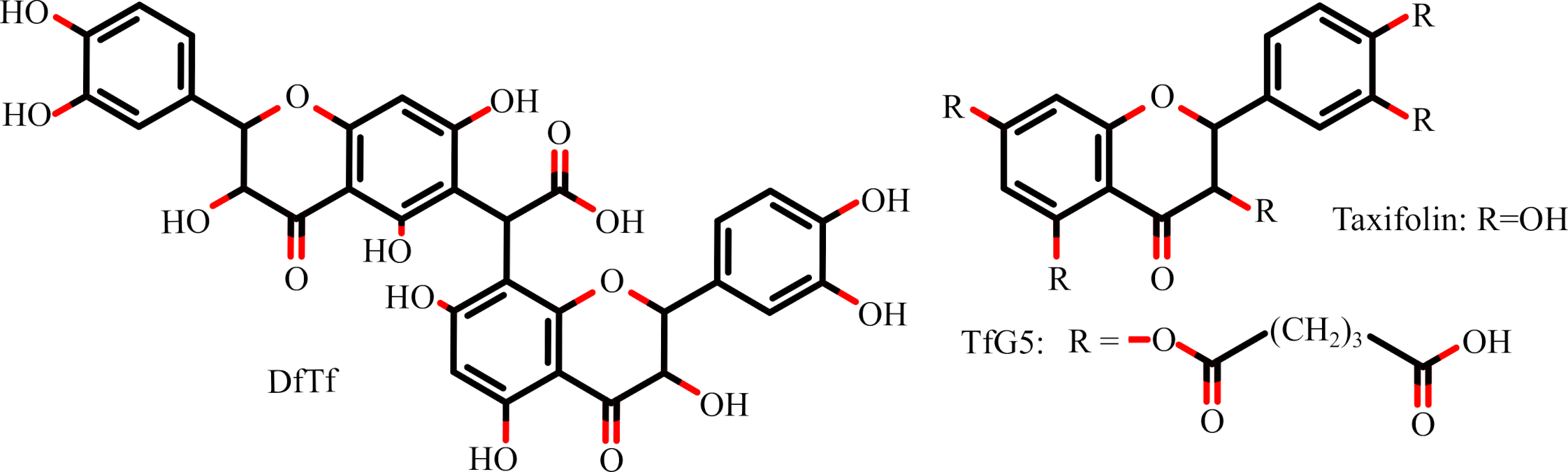

Supplement: Supplementary file 1 [file ijms-25-12353-s001.zip › fig1_v2.jpg]

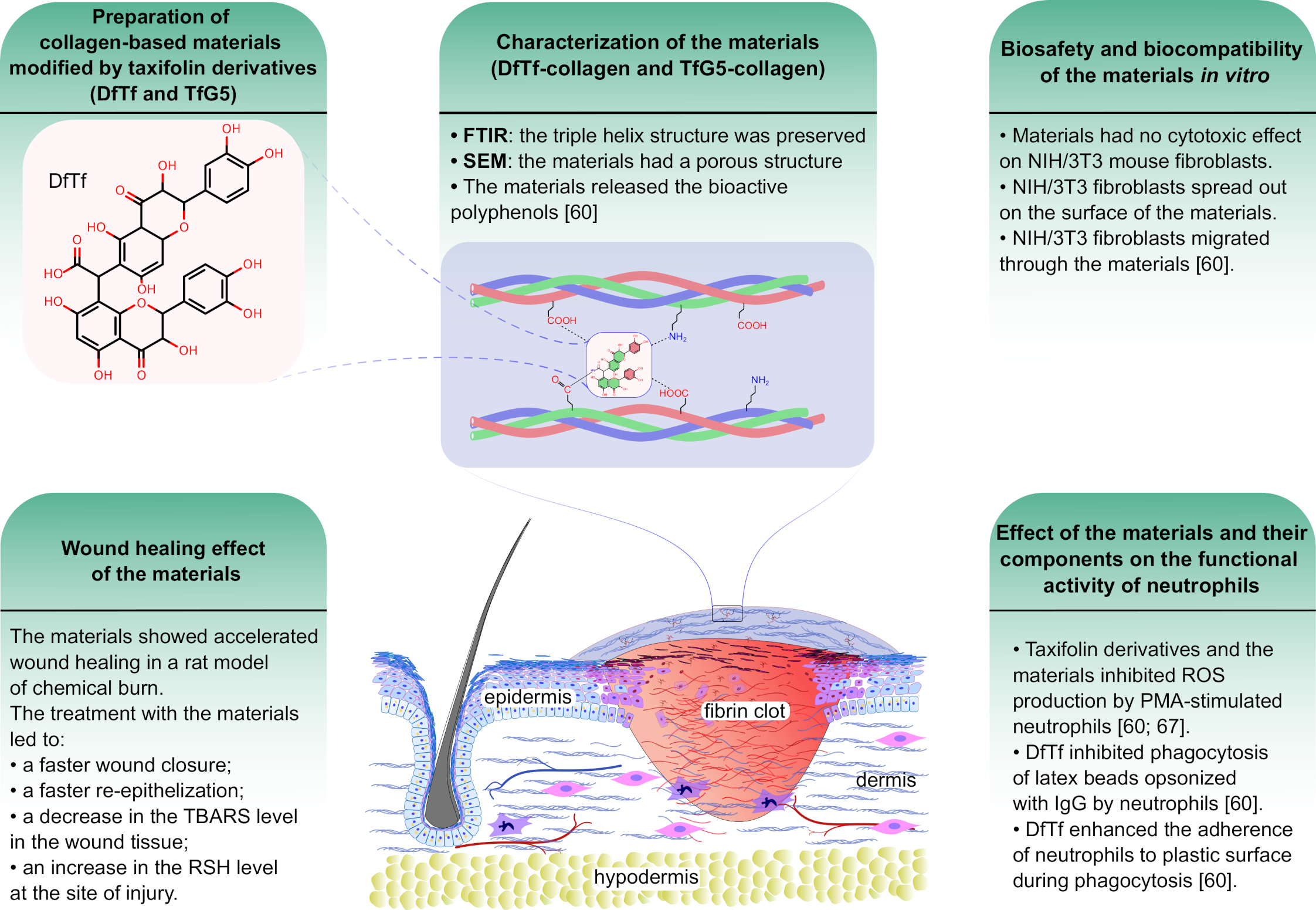

Supplement: Supplementary file 1 [file ijms-25-12353-s001.zip › fIg7_v2.jpg]

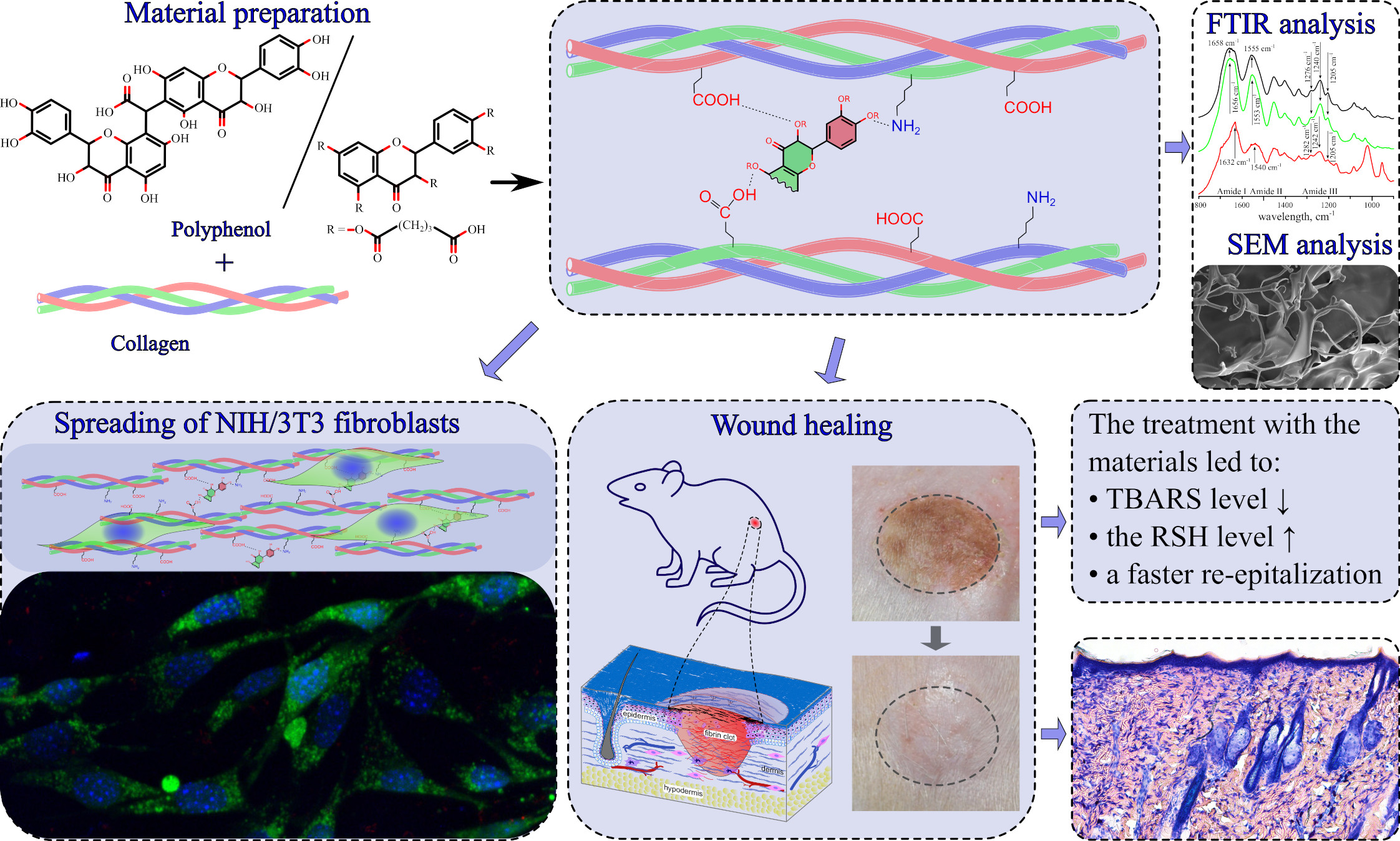

Supplement: Supplementary file 1 [file ijms-25-12353-s001.zip › GA_new12.jpg]
